# Looking for Possible Benefits of Combining Short-Chain Fructo-Oligosaccharides (scFOS) with *Saccharomyces cerevisiae* Sc 47 on Weaned Pigs Orally Challenged with *Escherichia coli* F4^+^

**DOI:** 10.3390/ani13030526

**Published:** 2023-02-02

**Authors:** Laia Ferreres-Serafini, Lorena Castillejos, Marga Martín, Cindy Le Bourgot, Susana M. Martín-Orúe

**Affiliations:** 1 Animal Nutrition and Welfare Service (SNIBA), Universitat Autònoma de Barcelona (UAB), 08193 Bellaterra, Spain; 2 Departament de Sanitat i Anatomia Animals, Universitat Autònoma de Barcelona (UAB), 08193 Bellaterra, Spain; 3 Tereos, R&D, 67390 Moussy-le-Vieux, France

**Keywords:** weaning piglet, *Escherichia coli* F4^+^, probiotic, prebiotic, synbiotic, fructo-oligosaccharides, *Saccharomyces cerevisiae*

## Abstract

**Simple Summary:**

Despite the use of antimicrobials in livestock being reduced in recent years, they are still widely used in swine production. These therapeutic products are commonly prophylactically used via feed or water to prevent disease and maintain productive indexes, particularly during critical periods when animals usually suffer from pathogen infections, such as post-weaning colibacillosis. However, there is an urgent need to avoid using these drugs due to increased antimicrobial resistance. Different probiotics and prebiotics are effective in preventing or limiting the disease, constituting possible alternatives. Still, there is scarce information regarding the potential benefits of combining both strategies through a synbiotic approach. In this study, we hypothesize that combining short-chain fructo-oligosaccharides (scFOS) and live yeast *Saccharomyces cerevisiae* Sc 47 could turn in an effective synbiotic to fight post-weaning colibacillosis in piglets due to complementary and/or synergistic effects. The objective of this work is to evaluate the effect of supplementing a blend of scFOS, live yeast *Saccharomyces cerevisiae* Sc 47, or their combination, on the health of weanling pigs orally challenged with enterotoxigenic *Escherichia coli* F4^+^. Our results show that these products’ mechanism of action could be slightly different; therefore, a beneficial effect could be expected from their synbiotic supplementation.

**Abstract:**

The objective of this work was to evaluate the effect of supplementing short-chain fructo-oligosaccharides (scFOS) combined or not with live yeast *Saccharomyces cerevisiae* Sc 47 on weanling pigs challenged with *Escherichia coli* F4^+^. We allocated ninety-six piglets to four experimental diets: control (CTR); supplemented with scFOS (5 g/kg Profeed^®^ P95) (scFOS); *S. cerevisiae* Sc 47 (1 g/kg Actisaf^®^ Sc 47 HR +) (YEA); or both (SYN). Parameters included: performance; *E. coli* F4^+^ detection; fermentation activity; inflammatory biomarkers; and ileal histomorphology. Our results showed that supplementing scFOS was able to reduce the incidence of diarrhea, and both supplements were able to lower counts of EHEC along the gut. Supplementing scFOS was mostly associated with changes in the gut ecosystem and increases in the lactobacilli population, while *S. cerevisiae* Sc 47 registered increases in the numbers of ileal intraepithelial lymphocytes. The synbiotic mixture showed the lowest diarrhea incidence and fecal scores, benefiting from complementary modes of action and possible synergistic effects due to a hypothesized yeast–LAB cross-feeding phenomenon in the foregut. In conclusion, our results evidence that supplementing scFOS or *Saccharomyces cerevisiae* Sc 47 is efficacious to fight post-weaning colibacillosis, and combining both could be beneficial in high-risk scenarios.

## 1. Introduction

In the modern swine industry, weaning has become the main critical period piglets have to overcome. This stage is characterized by sudden dietary, environmental, and social changes [1] that turn into new stressors for the piglet. Altogether, weaning can lead to a poorer immune response, an impairment of intestinal barrier function, and an unbalanced gut microbiota [2]. As the intestine plays a primordial role in protecting the animal from harmful microorganisms, post-weaning piglets become more susceptible to opportunistic pathogens [3]. *Escherichia coli* F4^+^ (*E. coli* F4^+^) is one of the pathogens frequently causing post-weaning colibacillosis [4,5], an infection characterized by the presence of watery feces, poor growth performance, and increased morbidity and mortality in piglets [6,7]. Until now, antibiotics has been widely used to control these incidences [8]; however, the increase in the amount of antimicrobial resistant pathogens creates an urgent need to find alternative strategies [9,10]. Furthermore, in some countries, the prophylactic and/or therapeutic use of antibiotics has been banned or highly restricted [11,12].

Some compounds, such as probiotics and prebiotics, have been gaining prominence in the feed industry as a new dietary strategy due to their potential antimicrobial and growth-promoting effects on animals [9,13,14]. Probiotics are defined as live microorganisms that confer a health benefit on the host when administered in adequate amounts [15]. Within the most frequently studied microorganisms with this potential, we can find: *Lactobacillus*, *Bifidobacterium*, *Enterococcus*, *Bacillus*, and yeast, such as different species of *Saccharomyces* [16]. Yeasts, from the genus *Saccharomyces*, have been demonstrated to improve gut integrity, strengthen the immune system, and enhance small intestine development in weaned piglets [6,17]. In addition, in-feed supplementation of *Saccharomyces* in weaned piglets has been suggested to decrease enterotoxigenic *E. coli* F4^+^ adhesion to the intestinal mucosa, in addition to this pathogen’s jejunal and cecum populations [17]. Prebiotics are defined as a substrate that is selectively utilized by host microorganisms to confer a health benefit [18,19]. Polymers such as fructo-oligosaccharides (FOS) and galacto-oligosaccharides (GOS) belong to this group of prebiotic ingredients [20] and are associated with the best documented benefits [21]. They promote the growth and activity of specific favorable bacteria and help in the maintenance of an optimal gut environment [20,22]. Differences have been reported in the prebiotic activity of FOS depending on the oligomer chain length and composition. Short-chain FOS (scFOS) promote a quicker growth of beneficial bacteria and short-chain fatty acid (SCFA) production compared with long-chain prebiotics, such as oligofructose or inulin [23]. In this study, we tested a source of scFOS produced from sucrose presenting a low degree of polymerization (DP 3-5).

Combining probiotics and prebiotics, generally referred to as synbiotics, has been proposed to maximize the benefits of both strategies [18]. According to the recent ISAPP definition for the term, a synbiotic is a mixture comprising live microorganisms and substrate(s) selectively utilized by host microorganisms that confers a health benefit on the host [24]. Benefits can be attributed to the complementary effects of both (complementary synbiotics), as well as the selective growth of the probiotic strain promoted by the prebiotic that is chosen to specifically stimulate its growth and activity (synergistic synbiotics) [24,25].

In this study, we hypothesize that combining short-chain fructo-oligosaccharides (scFOS) and live yeast *Saccharomyces cerevisiae* Sc 47 could turn in an effective synbiotic to fight post-weaning colibacillosis in piglets due to complementary and/or synergistic effects. The objective of the study was therefore to test the beneficial effects of including scFOS, *S. cerevisiae* Sc47, or their combination, in the diets of weanlings orally challenged with *E. coli* F4^+^, analyzing their impact on performance, clinical diarrhea, *E. coli* F4+ numbers, fermentation activity, inflammatory biomarkers, and ileal histomorphology.

## 2. Materials and Methods

The study was performed at the Experimental Unit of the Universitat Autònoma de Barcelona (UAB) with prior approval from the institution’s Animal and Human Experimental Ethical Committee (permit no. CEAAH 4026; DMAH 10118). The treatment, management, housing, husbandry, and slaughtering conditions conformed to European Union Guidelines (Directive 2010/63/EU).

### 2.1. Animals, Housing, and Experimental Design

The trial was organized as a level 2 high-risk biosecurity procedure, and all involved personnel received appropriate training.

A total of 96 21-day-old male piglets (Large White × Landrace × Pietrain) weighing 5.7 (±0.20) kg were used. All of the animals came from a high-sanitary-status farm in which mothers were not vaccinated against *E. coli*. Piglets were selected from 24 sows previously confirmed to be homozygous for the Mucin 4 (MUC4) gene, a factor related to a greater susceptibility to infections caused by *E. coli* F4^+^ [26]. Animals were transported to the UAB’s facilities and placed in four rooms of 8 pens each (32 pens, with 3 animals per pen). Each pen (3 m^2^) was partially slatted and had a feeder and a nipple drinker to provide food and water ad libitum. Each pen was provided with a plastic toy specifically designed for pigs of this age. The weaning rooms were equipped with automatic heating and forced ventilation. The experiment was carried out during the spring season (May–June).

The trial was conceived as a 2 × 2 factorial design (receiving or not probiotics × receiving or not prebiotics) with 4 experimental diets: control (CTR), pigs receiving a pre-starter basal diet; prebiotic (scFOS), pigs receiving short-chain fructo-oligosaccharides; probiotic (YEA), pigs receiving *Saccharomyces cerevisiae* Sc47; and prebiotic + probiotic (SYN), pigs receiving both. Upon arrival, the animals were distributed among rooms and pens according to their initial body weight to ensure a similar average within pens. The four experimental treatments were balanced within rooms (2 pens/treatment in each room). The number of replicates for each experimental diet was eight, considering the pen as the experimental unit. All of the groups were orally challenged with the pathogenic *E. coli* F4^+^ strain one week after arrival. Animals received the experimental diets during 15 experimental days.

### 2.2. Diets and Tested Products

The tested prebiotic was short-chain fructo-oligosaccharides (scFOS; Profeed^®^ P95; Beghin-Meiji) obtained from sugar beet sucrose through an enzymatic reaction. It is composed of a terminal glucose molecule (G) linked to fructose molecules (F) by a β1–2 bound, with 37 ± 6% 1-kestose (GF2), 47 ± 6% nystose (GF3), and 16 ± 6% 1F-β-fructofuranosyl nystose (GF4), and therefore presents a low degree of polymerization comprised between 3 and 5 (DP 3-5). It was included at a concentration of 0.5% (5 g/kg) in the scFOS and SYN diets. The tested probiotic strain was *Saccharomyces cerevisiae* Sc 47 (Actisaf^®^ Sc 47 HR+; Phileo Lesaffre). It was included at a concentration of 0.1% (1 g/kg) in the YEA and SYN diets according to the manufacturer’s instructions.

The basal diet was formulated and manufactured in a mash form to satisfy the nutrient requirement standards for pigs of this age (National Research Council (NRC), 2012). Details regarding ingredient and chemical composition are shown in Table 1. The basal diet was manufactured in 2 batches of 500 kg. The prebiotic was included in one of the mixer batches (batch 2) by adding 2.5 kg over 500 kg of basal diet. All of these procedures were performed in a feed mill (Pinsos Molinet SL; Gaià, Barcelona). The probiotic was subsequently included in 150 kg of plain basal diet (batch 1) and 150 kg of basal diet with prebiotic (batch 2) by using a small mixer in the UAB’s facilities (Servei de Granges i Camps Experimentals SGiCE) just before starting the trial.

### 2.3. Bacterial Strain

Piglets were orally challenged with an *Escherichia coli* F4^+^ strain isolated from the feces of a 14-week-old pig and provided by the Infectious Diseases Laboratory of the UAB (Ref. COLI30/14-3). This strain presented virulence factors F4ab, F4ac, LT, STb, and EAST1. It was negative for factors K99, F6, F18, F41, STa, VT1, VT2, and EAE.

Oral inoculums were prepared after 24 h of incubation at 37 °C in LB medium (Luria broth) with slow agitation (250 rpm) in an orbital incubator. The final concentration of the inoculum was 3 × 10^9^ colony forming units (CFU)/mL, determined by plate counting in Luria agar. Plates were seeded just before inoculating the animals. A total volume of 6 mL was orally administered to each animal by using a disposable syringe without a needle, reaching a dose of 1.8 × 10^10^ CFU/animal, to ensure an effective infection.

### 2.4. Experimental Procedure

Piglets were transferred from the commercial farm to the experimental unit at 21 days of age. Upon arrival, the animals were weighed, identified with ear tags, and distributed in pens according to their weight.

After an adaptation period (day 8), 6 mL of *E. coli* F4^+^ inoculum was orally administered to all the animals. To ensure that the stomach was full at the time of inoculation, facilitating bacterial colonization and standardizing conditions for all the animals, we removed food the same day early in the morning and replaced it 15 min before inoculation (approximately from 9 a.m. until 2 p.m.).

From the challenge onward, the animals were examined each day for clinical signs to assess their post-inoculation (PI) status (e.g., dehydration, apathy, and diarrhea). The fecal samples for microbiological analysis were taken from the heaviest pig in each pen (n = 32) on arrival day (0), and from the same animal before inoculation at day 8 (0PI), 12 (4PI), and 16 (8PI). The rectal temperature was assessed one day before the challenge and on days 1, 2, and 3PI. Individual fecal scores were registered on days 0, 1, 2, 3, 5, and 7PI by tactile fecal stimulation/sampling and/or visual inspection. The fecal score was evaluated using a 5-point scale (1: hard and dry feces; 2: well-formed firm feces; 3: formed feces; 4: pasty feces; 5: liquid diarrhea). Diarrhea was considered to be indicated by a score ≥4.

Performance was monitored throughout the experimental period. The piglets were weighed on days 0, 4, and 8PI, and the feed consumption was recorded on days 0, 1, 2, 3, 4, 7, and 8PI. The mortality rate was also noted. No antibiotic treatment was administered to the animals during the experiment.

At day 4PI (day 12) and 8PI (day 16), the intermediate- and the high-weight piglets from each pen (from the beginning of the trial) were euthanized, respectively. The animals were sequentially sampled during the morning (between 9:00 and 14:00 h). The remaining animals were euthanized at the end of the study. For that, animals were first sedated with the synergic effect of 2 mg/kg xylazine (Xilagesic; Les Franqueses del Vallès) plus 20 mg/kg ketamine (Ketamidor; Wels, Austria) IM. Blood samples were taken from the cranial cava vein, and then the animals were euthanized with an intravenous injection of 200 mg/kg sodium pentobarbital (Euthasol; Le Vet B.V., Oudewater, The Netherlands). The abdomen was immediately opened, and the digestive package was excised and transferred to a tray. Different samples were collected for analysis. The digestive contents of the ileum (considered as the distal 1/3 of small intestine) and full colon were emptied and homogenized, and their pH was measured. Subsequently, different aliquots of ileum and colon digesta were preserved for future analysis. For SCFA and lactic acid analysis, the samples were immediately frozen using dry ice and subsequently stored at −20 °C until analysis. For the ammonia analysis, 3 mL of colonic contents were collected with 3 mL of 0.2 N H_2_SO_4_ as a preservative solution. Subsequently, the sample was stored at −20 °C. For microbiological analysis, ileum and colon contents were collected in sterile containers, and ileum scrapings were placed in Eppendorfs before being stored at 4 °C until analysis. For the quantification of *E. coli* F4^+^ by quantitative PCR (qPCR), colon digesta aliquots and ileum scrapings were also collected and immediately frozen using dry ice, and stored at −20 °C. For the histological study, 2–3 cm-long sections of the distal ileum were cut, thoroughly washed with sterile PBS, and fixed by immersion in a solution of formaldehyde (4%).

### 2.5. Analytical Procedures

#### 2.5.1. Feed Analysis

Chemical analyses of the diets, including the dry matter, ash, crude protein, diethyl-ether extract, and crude fiber, were carried out according to the standard procedures of the Association of Official Agricultural Chemists (AOAC International, 1995). The gross energy of the diets was also determined on an adiabatic pump calorimeter.

#### 2.5.2. Microbiological Analysis

Lactobacilli and entero-hemorrhagic *E. coli* (EHEC) were measured by plate counting in selective MRS media (CM0361B MRS Agar OXOID) and selective chromogenic media (413697 CHROMIC- EHEC 6 × 200 mL Biomerieux), respectively.

*E. coli* F4^+^ was quantified in colonic digesta and ileum scrapings by qPCR using SYBR green dye. DNA from the colonic digesta and ileum scraping samples was extracted and purified using the commercial QIAamp Fast DNA Stool Mini Kit (Qiagen; West Sussex, UK). The DNA was eluted in 200 mL of Qiagen buffer AE and stored at −20 °C until use. Afterwards, a qPCR targeting the gene coding F4 fimbria was performed using the SYBR green dye according to the procedure described by Hermes et al. (2013) [27].

#### 2.5.3. Serum Analyses

The blood samples were centrifuged (2500× *g*, 8 min at 4 °C) after 4 h of refrigeration, and the serum obtained was divided into different aliquots before storage at −20 °C.

The concentrations of Tumor Necrosis Factor-α (TNF-α) and major acute-phase protein (PigMAP) in the serum were determined by ELISA (Quantikine Porcine TNF-α kit, R&D Systems; Minneapolis, MN, USA) and a sandwich-type ELISA (Pig MAP kit ELISA, Pig CHAMP Pro Europe S.A.; Segovia, Spain), respectively, following the manufacturer’s recommendations.

#### 2.5.4. Short-Chain Fatty Acids, Lactic Acid, and Ammonia Analyses

The concentrations of short-chain fatty acids (SCFAs) in the colonic digesta and feces were analyzed by gas chromatography, after which the samples were subjected to an acid–base treatment followed by an ether extraction and derivatization with N-(tertbutyldimethylsilyl)-N-methyl-trifluoroacetamide (MBTSTFA) plus 1% tertbutyldimethylchlorosilane (TBDMCS) agent using Richardson et al.’s method (1989) [28] modified by Jensen et al. (1995) [29].

The ammonia concentrations on H_2_SO_4_-preserved colonic digesta samples were assessed by using a gas-sensitive electrode (Hatch Co.; Loveland, CO, USA) combined with a digital voltmeter (Crison GLP 22, Crison Instruments, S.A.; Barcelona, Spain). For that, the preserved samples were homogenized and centrifuged at 1500× *g* for 10 min before the analysis, and diluted with distilled water (1:2 and 1:4 according to the ammonia concentration), and 10M NaOH was added to ensure a final pH > 11. Once the ammonia was released, it was measured in the supernatants as the voltage change (in mV).

#### 2.5.5. Histological Analysis

Morphological measurements of ileal sections were performed with a light microscope (BHS, Olympus; Barcelona, Spain) following Nofrarias et al.’s method (2006) [30]. Measured parameters included: villus height (VH), crypt depth (CD), ratio villus:crypt (VH:CD), intraepithelial lymphocytes (IEL), goblet cells (GC), and mitosis (M). We measured between 7 and 10 intact villi per animal.

#### 2.5.6. Mother Genotyping for *E. coli* F4^+^ Susceptibility

The Mucin 4 (MUC4) gene is proposed as one of the genetic markers for pig *E. coli* F4^+^ resistance/susceptibility [4,26]. To assure the susceptibility of piglets in this trial, the mothers were genotyped for this gene polymorphism. Hair follicles were collected from 72 mothers to extract the DNA, following the procedure described by Luise et al. (2019) [26]. A restriction fragment length polymorphism analysis (PCR-RFLP) was performed following the method described by Jørgensen et al. (2003) [31]. Piglets were selected from 24 sows homozygous for the susceptible gene (MUC4^GG^).

### 2.6. Statistical Analysis

The effects of the experimental diets on all the data were analyzed using the free R statistical analysis software version x64 4.0.3 (R Development Core Team; Franklin Lakes, NJ, USA) using the stats package [32].

The results are expressed as means with their standard errors unless otherwise stated. The microbiological data were previously log transformed.

Most of the data were analyzed by a two-way analysis of variance (lme function) to determine the main effects of prebiotic addition (PRE), probiotic addition (PRO), or any possible interaction (PRE × PRO). Analyses were carried out using a generalized linear model as follows:

Y_ijk_ = μ + PRE_i_ + PRO_j_ + (PRE × PRO)_ij_ + e_ijk_

where Y is each observation of the studied variable, μ is the global mean, PRE_i_ is the main effect of adding the prebiotic to the diets, PRO_j_ is the main effect of adding the probiotic to the diets, and PRE x PRO_ij_ is the possible interaction. Finally, e_ijk_ is the experimental error term.

When the effect of the interaction (PRE × PRO) was established (*p* = 0.05), the means were compared using the probability function of differences adjusted by the Tukey–Kramer method.

For an analysis of the evolution of LW, ADG, ADFI, and fecal scores along time, a mixed-effects model (lme function) was used following:

Y_ijk_ = μ + Diet_i_ + Time_j_ + (Diet × Time)_ij_ + e_ijk_

where Y is each observation of the studied variable, μ is the global mean, Diet_i_ is the main effect of the experimental diets, Time_j_ is the main effect of the day/period, and Diet × Time_ij_ is the possible interaction. Finally, e_ijk_ is the experimental error term.

The mortality percentage and *E. coli* F4^+^ prevalence were analyzed by means of frequency analysis using a Fisher test (fisher.test function) with the same statistical package.

The score fecal data in were analyzed using a non-parametric approach, namely the Kruskal–Wallis test (kruskal.test) [33]. When the effect of treatment was established (*p* = 0.05), the means were compared using Dunn’s test (dunnTest) with the FSA package [34].

For all of the parameters, the pen was considered as the experimental unit. The α level used to determine statistical significance was *p* = 0.05. The statistical trend was considered for *p* > 0.05 and *p* < 0.10.

## 3. Results

In general terms, the trial developed as expected without any remarkable incidents. Upon arrival, all animals showed a good health status, and no presence of *E. coli* F4^+^ was detected in feces before inoculation. After the oral inoculation, the animals showed a mild course of diarrhea that spontaneously resolved at the end of the study without the need of any pharmacological treatment. During the PI period, three pig deaths were recorded: one from the CTR group at day 2PI and two from the SYN group at days 2PI and 3PI.

One pen from the CTR group was excluded from the study and treatment due to negligible feed intake along the first week. Average daily feed intake in this pen during the first week was 18.6 g/day.

### 3.1. Performance Parameters

Average values of live weight (LW), average daily gain (ADG), average daily feed intake (ADFI), and gain-to-feed ratio (G:F) are presented in Table 2.

No significant differences due to treatments were found in LW at the beginning or end of the study. No remarkable differences were found in ADG, despite a trend in the PRO group presenting lower values during adaptation week. However, ADFI showed differences between treatments after the challenge with a lower feed intake in those animals receiving the probiotic (YEA and SYN treatments). The gain-to-feed ratio (G:F) did not show significant differences related to the experimental treatments.

### 3.2. Clinical Signs

**Figure 1:**
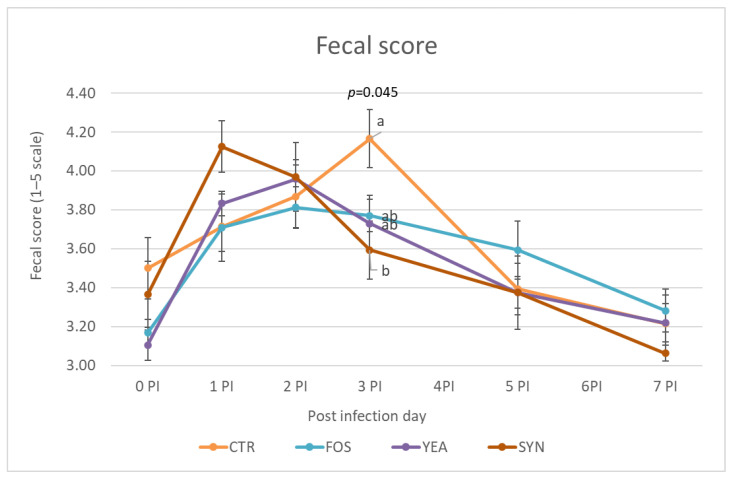
Evolution of mean fecal score in different experimental groups during post-inoculation period. CTR (in orange): animals receiving pre-starter basal diet; scFOS (in blue): animals receiving basal diet with short-chain fructo-oligosaccharides supplementation; YEA (in purple): animals receiving basal diet with *Saccharomyces cerevisiae* Sc 47 supplementation; SYN (in red): animals receiving both supplementations (scFOS + YEA). N = 7 for CTR group, N = 8 for rest of groups. ^a,b^ indicate statistically significant differences between groups.

Fecal scores were measured from the inoculation day until one week later (Figure 1). At day 0PI, the mean fecal score for the different treatments was around 3–3.5. Once inoculated, the animals’ fecal score worsened, with increased scores up to day 3PI and decreasing afterwards. At day 3PI, the SYN treatment showed significantly lower (better) scores than the CTR group, while scFOS and YEA presented intermediate values.

Table 3 shows the changes induced by the treatments in the incidence of diarrhea determined as the percentage of animals per pen with fecal scores ≥4. After one week of adaptation and just before the challenge (day 0PI), there was a significant PRE × PRO interaction (*p* = 0.001), resulting in CTR and SYN groups showing higher diarrhea prevalence. Treatments only including the probiotic or prebiotic showed the lowest levels of diarrhea. One day after the challenge (day 1PI), animals receiving the probiotic showed significantly higher diarrhea incidence, regardless of whether or not they were receiving the prebiotic (66.3% vs. 35.3%, respectively, *p* = 0.006). However, at day 3PI, animals receiving the prebiotic registered a lower diarrhea rate (34.1% vs. 55.2%, *p* = 0.035), and there was also a trend for a lower incidence with the probiotic (36.3% vs. 52.9%, *p* = 0.083). SYN treatment benefits from an additive effect (no interaction), resulting in the lowest numerical values.

The rectal temperature of each animal was recorded at 0, 24, 48, and 72 h PI. Rectal temperature showed normal values throughout the study, with no fever detected (>40.5). No significant differences due to treatments were registered after the oral challenge. However, on day 0PI (before inoculation), supplementing the prebiotic was associated with significantly lower temperatures (38.8 vs. 39.1, *p* = 0.032), which was a similar trend to the probiotic (38.8 vs. 39.1, *p* = 0.082).

### 3.3. Microbiological Analysis

Fecal plate counts of EHEC on selective chromogenic media (Table 4) showed no growth before the inoculation. However, after the challenge (day 4PI), plate counts reached high values. At day 4PI, animals receiving the prebiotic showed higher fecal counts regardless of whether or not they were receiving the probiotic (9.20 vs. 7.76 CFU/g, respectively, *p* = 0.048), and no effect was found related to probiotic addition. However, at day 8PI, there was a significant interaction, and all supplemented diets showed lower counts compared with CTR, despite no additive effect for the combined treatments (SYN) being found.

Contrary to feces, plate counts of mucosa scrapings at day 4PI showed that animals receiving the prebiotic exhibited lower numbers of EHEC (5.54 vs. 7.34 CFU/g, *p* = 0.002), with no impact for probiotic supplementation. At day 8PI, and similarly to feces, there were no significantly lower values for the three supplemented diets compared with the CTR diet. Ileum digesta samples also showed similar reductions for the three supplemented diets at day 8PI, with the combination of the prebiotic and probiotic exhibiting an additive decrease in EHEC (SYN diet); therefore, the interaction was not significant. For colon digesta, significant decreases were also found related to the prebiotic or probiotic supplementation at both 4PI and 8PI, but without additive effects.

In relation to fecal plate counts of lactobacilli in selective MRS medium (Table 4), no differences due to treatments were seen before the inoculation (day 0PI) or on day 4PI. However, samples collected on day 8PI showed higher counts of lactic bacteria with the supplemented diets; however, no additive effect was found for the SYN combination (significant interaction). Plate counts of mucosa scrapes on day 8PI showed the clear impact of supplementing the probiotic on lactobacilli counts, with a trend for higher values when supplemented alone (interaction *p* = 0.083). Counts of ileal and colonic digesta did not show significant differences due to treatments in any of the sampling days.

When the microbiological results were also expressed as the log ratio of lactobacilli/EHEC (Table 4), in most cases, there was a clear significant effect of supplementing the prebiotic or the probiotic, namely higher ratios; however, there were no additional effects when combining both ingredients.

### 3.4. Escherichia coli F4^+^ qPCR Quantification

The results of qPCR quantification showed that not all the animals obtain quantifiable amounts of F4 genes after the challenge. Regarding colonic digesta, eight animals showed non-quantifiable values (five at day 4PI and three at day 8PI), while in ileal scrapings, nine animals showed non-quantifiable values at day 8PI. No differences between treatments were found in the number of animals with non-quantifiable *E. coli* F4^+^.

Table 5 shows *E. coli* F4^+^ numbers in ileal scrapings and colonic digesta samples (log F4 genes copies/g FM) only considering quantifiable animals. As expected, the numbers decreased from day 4 to 8PI in both types of samples; however, no statistical differences were found between treatments, except a trend for the interaction in ileal scrapings at day 8PI, where higher values for all the supplemented diets were observed.

### 3.5. Microbial Fermentative Activity

Regarding pH values and ammonia concentration in colonic digesta samples, no significant differences were found related to the experimental treatments.

Table 6 shows SCFA concentrations and their molar proportions, measured in colonic digesta at day 4PI and 8PI and in fecal samples at day 0PI (day 8) and 8PI (day 16). Colonic digesta samples showed lower SCFA concentrations at day 8PI with the combination of the pre- and probiotic (SYN diet) when compared with other supplemented diets (*p* interaction = 0.096). This treatment was also associated with the highest molar proportions of BCFA (branched-chain fatty acids) (*p* interaction = 0.026). In fecal samples, SCFA concentrations also showed lower values at day 8PI when both ingredients were combined (*p* interaction = 0.023), but in this case without significant changes in the molar proportion of BCFA. Statistical trends were found in the SYN group for molar proportions of propionate at day 0PI (*p* interaction = 0.057), which presented higher values compared with other treatments, and for molar proportions of valerate at day 0PI (*p* interaction = 0.085), which presented lower values than other groups.

### 3.6. Immune Response

The animals’ inflammatory response was assessed by measuring serological levels of TNF-α (pro-inflammatory cytokine) and Pig-MAP (main acute phase protein in the pig) at day 0PI (before inoculation), 4PI, and 8PI. The average values are detailed in Table 7.

In general terms, no significant effects were reported related to the experimental treatments, except for a trend for a reduction in TNF-α levels with the scFOS diet detected after the week of adaptation and just before the challenge (*p* interaction = 0.063).

### 3.7. Ileal Histomorphometry

Table 8 shows the average value for the different histomorphological parameters determined in ileal sections. No significant effects attributed to the experimental treatments were detected, except for a significant increase in the number of intraepithelial lymphocytes (IEL) at day 8PI when animals received the probiotic regardless of whether or not they received the prebiotic (17.17 vs. 13.97 cell n°, respectively, *p* = 0.046; 6.36 vs. 5.14 cell n°/100 µm, respectively, *p* = 0.009), as well as a significant increase in the number of mitotic cells (M) at day 8PI with probiotic supplementation (1.15 vs. 0.69 cell n°, *p* = 0.008; 0.61 vs. 0.36 cell n°/100 µm, *p* = 0.01).

## 4. Discussion

This trial aimed to expand our knowledge in the search for non-antibiotic alternatives to strengthen piglets in one of the most critical periods of their lives. To do so, we assessed the effectiveness of dietary supplementation based on a prebiotic or probiotic, as well as the beneficial impact of adding both as a potential synbiotic in *E. coli* F4^+^-challenged piglets [4,35,36].

Ensuring an effective infection is a crucial step in challenge models. In this trial, different parameters confirmed the successful infection of the animals. Clinical signs of moderate diarrhea, with a peak just after the inoculation, together with the qPCR detection of *E. coli* F4^+^ in mucosa scrapings and ileal and colonic digesta, proved the effectiveness of the inoculation. A similar moderate clinical response was observed in our previous studies using a similar model [27,37,38,39,40]. Despite qPCR failing to evidence favorable effects for the experimental diets in post-weaning colibacillosis, EHEC plate counts showed the considerable impact of all supplemented diets, with reductions in all intestinal sampling sites. Apparent discrepancies between both methods could be the result of differences in sensitivity and targets. The qPCR detection of F4 fimbria is a very specific methodology that can detect any *E. coli* strain bearing this gene. In this work, no F4 fimbria were qPCR detected before the inoculation; therefore, we could assume that qPCR values after the challenge corresponded to the inoculated strain. However, it cannot be fully excluded that another indigenous strain could have taken the opportunity to grow. Furthermore, the registered levels were very close to the method’s minimum level of detection (approximately 2.5 gene copies/g FM), and several animals presented not-quantifiable levels. Being so close to the minimum level of detection probably precludes detecting differences with this method.

Selective chromogenic media used for EHEC plate counts (CHROMICTM-EHEC Agar Biomerieux) were specifically designed for the selective isolation and presumptive identification of enterohemorrhagic *E. coli* (EHEC), including different serotypes. Our inoculated strain is able to grow on this medium; however, many other indigenous strains of EHEC could also grow. The EHEC plate count numbers were higher than those obtained by qPCR, even considering the different units used (CFU vs. gen numbers), suggesting that other EHEC strains had also been quantified. By inoculating the animals with the *E. coli* F4, we were able to induce colonization and infection of the gut with a presumable associated dysbiosis. Disrupting the ecological equilibrium of the gut could have favored the proliferation of other opportunistic pathogens sharing the same ecological niche with the inoculated *E. coli*. From this point of view, the EHEC plate count results not only reflect the impact of diets on the specific inoculated *E. coli* F4^+^, but also the growth of other indigenous EHEC strains.

Fructo-oligosaccharides (FOS) are attractive prebiotic fibers, which are suggested to improve weaned pigs’ intestinal morphology and growth performance, even after exposure to *E. coli* F4^+^. Short-chain fructo-oligosaccharides (scFOS), such as those tested in this study, have a lower degree of polymerization (between 3 and 5) when compared with other FOS or inulin (≥9 units). Short-chain FOS are known to be fermented more effectively and more quickly than other sources due to this lower degree of polymerization in their structure. Some in vitro studies have demonstrated that scFOS are more effective in stimulating SCFA production [41] and inducing an effective prebiotic effect than longer chains, such as oligofructose and inulin [42,43]. Previous studies have demonstrated that this source of scFOS is selectively fermented by gut microbiota, mainly promoting the growth of *Lactobacillus*, *Bifidobacterium*, and *Prevotella* at the expense of *E. coli* growth, and scFOS are also able to increase the concentration of SCFA [44,45,46,47,48,49]. Moreover, this type of scFOS has been associated with changes in the immune response and with the enhancement of intestinal architecture in neonatal and weaned piglets [44,46,47,50,51]. Other favorable outcomes reported with dietary scFOS supplementation in piglets include the modulation of inflammatory responses by downregulating some cytokines, such as TNF-α and IL-6 in the small intestine [48,52]. Until now, less was known about the effect of scFOS in pigs challenged with *E. coli* F4^+^.

In this trial, a reduction in diarrhea prevalence was seen in animals supplemented with scFOS. Reductions were detected at the end of first week, when scFOS was supplemented alone, and also shortly after the challenge (at day 3PI). One hypothesis that would explain reductions in diarrhea with scFOS would be the specific promotion of lactic acid bacteria, particularly *Lactobacillus* and *Bifidobacterium*, which have the ability to exclude opportunistic pathogens, such as *E. coli* [53,54]. Therefore, our results showed a promoting effect of scFOS on lactobacilli in feces at day 8PI, with reductions in EHEC plate counts in ileal scrapings and in ileum and colon digesta. As a result, the ratio of lactobacilli/EHEC was also lower in ileal and colonic digesta and in feces at day 8PI, as well as in ileal scrapings and colon digesta at day 4PI, proving the beneficial competition between bacteria populations exerted by scFOS supplementation. However, despite reductions in diarrhea with the prebiotic, we were not able to detect significant improvements on animal growth performance. Probably because of the limited number of replicates, the high individual variability observed in these challenge models, as well as the short period of scFOS supplementation, precluded us from finding statistical differences in performance indexes.

Supplementation with scFOS has been commonly associated with increases in SCFA [46,48]. In this trial, we also found an increase in the SCFA concentration in feces and the colon (a trend) at day 8PI, but only when scFOS was administered alone and not combined with the probiotic. These results would suggest that combining scFOS and *Saccharomyces cerevisiae* Sc 47 led to a decrease in the amount of fermentable carbohydrates (including scFOS) arriving to the hindgut. This possibility is also suggested by the higher molar proportions of BCFA registered in the colon at day 8PI. This reduction in the arrival of fermentable carbohydrates could be due to an increased fermentative activity at the end of the small intestine, as fermentation at the ileal level has been reported to be relevant in pigs [55]. A cross-feeding phenomenon between *S. cerevisiae* and lactic acid bacteria (LAB), in ileum in the presence of scFOS, could be behind such an effect. Ponomarova et al. [56] described a similar yeast–LAB cross-feeding community in the presence of lactose, in which *Saccharomyces cerevisiae* enabled the survival of *Lactobacillus plantarum* and *Lactococcus lactis* by secreting a pool of metabolites, especially amino acids, and LAB provided yeast with free glucose from lactose in a commensal association.

These effects, seen in microbial populations and fermentation activity with scFOS, were not associated with changes in ileal architecture, as had been previously reported in challenged piglets, in which the villus height and the ratio V/C were increased after FOS supplementation [57]. No effects were seen in the immunity indexes measured in this study (TNF-α, PigMap nor IEL), except a trend in TNA-α, which displayed a decrease at day 0PI when supplemented alone (in agreement with [48]). However, other authors have reported differences in cytokine and IgA secretion [44,48]. Altogether, our results suggest that scFOS reduced diarrhea, mainly through a preventive activity based on the growth promotion of beneficial bacteria that had competitively excluded potential opportunistic pathogens, such as *E. coli.*

Regarding the probiotic used in this trial, *Saccharamoyces cerevisiae* Sc 47, it has been previously documented to generate beneficial effects when tested in piglets challenged with *E. coli* F4^+^. Its favorable effects include reductions in diarrhea scores, *E. coli* F4^+^ attachment to intestinal mucosa, and fecal *E. coli* F4^+^ shedding, and improvements to the intestinal architecture [35,58,59,60,61]. Supporting the literature, at day 3PI, the probiotic-supplemented animals tended to present lower diarrhea prevalence (*p* = 0.083), which was similar to the reductions observed with scFOS. Lower diarrhea was also registered at the end of the first week when the probiotic was supplemented alone. However, in contrast to scFOS, animals receiving *S. cerevisiae* Sc 47 exhibited a considerable increase in diarrhea prevalence just one day after the pathogen inoculation (1PI). This could suggest the higher intestinal susceptibility of these animals to be colonized. This idea is in consonance with the reduction in ADFI registered with probiotic supplementation after the challenge, which was also seen in this and other trials [62]. Nevertheless, despite this hypothesized higher susceptibility, the administration of the probiotic could have enhanced the piglets’ response to the pathogen, which finally results in a good control of the infection. Considerably lower EHEC counts were found in the ileum, mucosa, colon, and feces of these animals, with significant increases observed in the lactobacilli/EHEC ratios. These results clearly emphasize an antagonistic effect exercised by the probiotic against *E. coli* F4^+^. In vitro, this antagonism has been explained by the nutrient competition between yeasts and bacteria, the modification of the physico-chemical conditions of the digestive environment, or by the yeast production of inhibitory substances, such as proteases or ethanol [63]. Other in vivo trials have also supported the beneficial effect of *Saccharomyces cerevisiae*, showing an increase in the lactobacilli population in intestinal contents [59]. Supporting this hypothesis, we also found increased levels of lactobacilli in feces and ileal scrapings at day 8PI.

Other evidence supporting the hypothesis of yeast improving the animal response against the pathogen comes from ileal histomorphometry. In accordance with Bontempo et al. [64] and Trevisi et al. [65], increases in mitotic cell counts were found in the ileum sections of the supplemented animals. Enterocytes are constantly being replaced in crypts, and the rate of replacement should match the rate of loss. During weaning, intestinal mucosa can become thinner due to different insults requiring more proliferating cells [64]. Therefore, our results would suggest that yeast-supplemented piglets had a better capacity to restore the intestinal mucosa architecture by increasing cell replacement; however, despite this, increased mitotic activity without changes in the villus/crypt ratio could also be regarded as an increased pathogen invasiveness resulting from the probiotic. Moreover, an increased number of intraepithelial lymphocytes was also seen in pigs receiving the yeast. Weaning stress has been associated with decreases in these cells and an increased apoptosis, and Bontempo et al. and Zhaxi et al. [64,66] reported that a greater number of lymphocytes in the intestine could represent an enhanced intestinal mucosa immunity. Taking this into account, the moderate increase observed in the IEL with the probiotic could be regarded as a positive effect.

The improved response of the piglets to the pathogen challenge with the probiotic could therefore be due to different mechanisms. On the one hand, supplementing *S. cerevisiae* Sc 47 could have caused ecological changes in the gut microbiota, as previously described by other authors [67], which was suggested by the changes observed in lactobacilli population. Particularly relevant is the increase observed in the number of lactobacilli in ileal scrapings, which could have resulted in an improved mucosa immunity in agreement with the observed increases in IEL. We also hypothesize that a boosted immune response could have been mediated by a directed effect of the probiotic, because it has been demonstrated to display multiple immuno-modulatory effects at the molecular level in intestinal porcine epithelial cells [68]. In short, the benefits of supplementing *S. cerevisiae* Sc 47 for the *E. coli* F4^+^ challenge seems to be mediated not so much by a preventive effect, such as scFOS, but by an improved response directly or indirectly mediated by the supplemented probiotic.

In this study, we assessed supplementing animals with a combination of scFOS and *S. cerevisiae* Sc 47 as a potential effective synbiotic for post-weaning colibacillosis [69]. The results showed that, at day 3PI, the greatest reduction in diarrheal clinical signs (fecal scores) was found with the SYN combination, reaching considerably lower values compared with CTR, supporting their effectiveness to fight *E. coli*. However, before the challenge, the combination of both additives did not result in any improvement compared with CTR, whereas the single supplements were shown to reduce the number of diarrheic pigs. These results would support the benefits of using this synbiotic combination in challenging scenarios, such as farms with a high incidence of post-weaning diarrhea; however, the benefits may be minimal in farms with high hygienic standards.

In summary, the way this synbiotic would help to fight post-weaning colibacillosis would rely on combining the different mechanisms of action discussed above for each of their components. Combining the preventive benefits from scFOS and a boosted animal response promoted by *S. cerevisiae* Sc 47 would explain why the SYN treatment was associated with the lowest EHEC counts and the highest ratio of lactobacilli/EHEC in the colon at day 8PI. Moreover, as discussed above, the combination of scFOS and *S. cerevisiae* Sc 47 could also have led to yeast–LAB cross-feeding phenomena, which synergistically promoted the growth of lactobacilli at the end of the small intestine, favoring the exclusion of *E. coli*.

## 5. Conclusions

Our results evidence that supplementing scFOS and *Saccharomyces cerevisiae* in the pre-starter diets can turn in an efficacious synbiotic to fight post-weaning colibacillosis in piglets. Their combined administration would benefit from complementary modes of action, including preventive effects based on beneficial changes in the intestinal ecosystem, particularly increasing the lactobacilli population, and improvements in the animal response in front of the pathogen, with increases in ileal IEL and mitotic activity. Our results also suggest a possible synergistic effect mediated by an hypothesized cross-feeding phenomenon between *S. cerevisiae* and lactic acid bacteria (LAB) in the small intestine. Combining both strategies could be particularly beneficial when animals have to face an elevated risk of post-weaning colibacillosis on the farm.

## Figures and Tables

**Table 1:** Ingredients and analyzed composition of basal diet (g/kg FM).

Ingredients	(g/kg FM)
Maize	205.50
Wheat	180.40
Barley 2 row	170.30
Extruded soybean	150.00
Sweet whey powder (cattle)	112.00
Fishmeal LT	60.00
Soybean meal 47	70.00
Whey powder 50% fat	25.00
Monocalcium phosphate	6.50
Calcium carbonate (CaCO_3_)	3.80
L-Lysine HCL (78)	4.50
Vit min GPlus (Balsa) *	4.00
Sodium chloride	2.40
DL-Methionine 99	2.60
L-Threonine	2.30
L-Tryptophane	0.70
**Analyzed Chemical Composition (%)**	
Dry matter	91.2
Ash	4.98
Crude protein	18.6
Crude fiber	1.86
EE	6.55

FM, fresh matter. LT, low-temperature preparation of fishmeal to preserve amino-acid content. EE, ether extract. * Provided per kilogram of complete diet: 12,000 IU vitamin A; 1400 IU vitamin D3; 55 UI vitamin E; 2 mg vitamin K3; 3 mg vitamin B1; 7 mg vitamin B2; 7.33 mg vitamin B6; 0.06 mg vitamin B12; 17 mg calcium pantothenate; 45 mg nicotinic acid; 0.2 mg biotin; 1.5 mg folic acid; 60 mg Fe; 90 mg Cu; 95 mg Zn; 40 mg Mn; 0.7 mg I; and 0.3 mg Se.

**Table 2:** Effect of experimental treatments on animal performance. LW (g), ADFI (g/day), ADG (g/day), and G:F for adaptation period (days 1–7), first post-inoculation (PI) period (days 0–4PI), second PI period (days 4–8PI).

Main Effects	PRO−		PRO+			*p*-Value		
	PRE−	PRE+	PRE−	PRE+	RSE	PRE	PRO	Interaction
**Diets**	**CTR**	**scFOS**	**YEA**	**SYN**				
**LW (g)**								
D0	5659	5662	5643	5657	116.7	0.842	0.802	0.896
D15	7905	7671	7613	7795	720.5	0.921	0.752	0.438
**ADFI (g/day)**								
adapt.	308	347	310	300	80.54	0.430	0.294	0.674
0–4PI	257	214	211	178	60.38	0.182	**0.038**	0.591
4–8PI	416	386	347	314	70.19	0.439	**0.005**	0.740
**ADG (g/day)**								
adapt.	79.8	83.6	62.2	63.1	29.63	0.830	0.091	0.896
0–4PI	108.2	58.9	48.1	43.0	95.89	0.649	0.164	0.342
4–8PI	340.7	311.7	351.7	359.1	143.80	0.831	0.889	0.445
**G:F**								
adapt.	0.294	0.230	0.202	0.226	0.1028	0.621	0.247	0.281
0–8PI	0.354	0.297	0.330	0.405	0.1291	0.856	0.391	0.183
total	0.233	0.205	0.222	0.259	0.0646	0.847	0.382	0.197

PRO+/−: presence/absence of probiotic ingredient in diet. PRE+/−: presence/absence of prebiotic ingredient in diet. N = 7 for PRE− and PRO− groups, N = 8 for rest of groups. RSE, residual standard error.

**Table 3:** Treatment effect on percentage of animals with diarrhea per pen measured at day 0, 1, 2, 3, 5, and 7 post inoculation. Considering diarrhea as a score ≥ 4.

Main Effects	PRO−		PRO+			*p*-Value		
	PRE−	PRE+	PRE−	PRE+	RSE	PRE	PRO	Interaction
**Diets**	**CTR**	**scFOS**	**YEA**	**SYN**				
**% Diarrhoea/Pen**								
0PI	37.7 ^a^	8.3 ^b^	8.3 ^b^	37.1 ^a^	20.81	0.969	0.969	0.001
1PI	37.7	33.1	57.9	74.6	28.54	0.559	0.006	0.308
2PI	57.0	53.8	70.6	49.6	34.08	0.332	0.702	0.476
3PI	71.0	37.1	41.4	31.1	27.54	0.035	0.083	0.244
5PI	9.4	26.9	16.5	12.4	20.87	0.463	0.727	0.206
7PI	14.1	12.4	8.25	0	16.91	0.418	0.145	0.599

PRO+/−: presence/absence of probiotic ingredient in diet. PRE+/−: presence/absence of prebiotic ingredient in diet. N = 7 for PRE− and PRO− groups, N = 8 for rest of groups. ^a,b^ indicate statistically significant differences between groups. PI, post inoculation; RSE, residual standard error.

**Table 4:** Plate counts (log CFU/g) of EHEC (chromogenic media), lactobacilli (selective MRS medium), and ratio of lactobacilli/EHEC (as difference of logarithms) in fecal, ileal mucosa scrapings, and ileum and colon digesta samples. Values are given for day 4 and 8 post inoculation.

Mains Effects		PRO−		PRO+			*p*-Value		
		PRE−	PRE+	PRE−	PRE+	RSE	PRE	PRO	Interaction
**Diets**		**CTR**	**scFOS**	**YEA**	**SYN**				
**EHEC**	**Day**								
Faeces	4PI	7.75	9.91	7.77	8.49	1.939	0.048	0.329	0.310
	8PI	6.28 ^a^	4.88 ^b^	4.70 ^b^	5.01 ^b^	0.791	0.064	0.017	0.006
Ileal mucosa	4PI	7.96	5.61	6.80	5.46	1.505	0.002	0.235	0.359
	8PI	5.43 ^a^	4.14 ^b^	4.22 ^b^	4.50 ^b^	0.454	0.004	0.014	<0.001
Ileal digesta	4PI	7.79	5.45	5.48	6.24	1.932	0.267	0.285	0.034
	8PI	5.42	4.27	4.41	3.98	0.616	0.001	0.007	0.119
Colonic digesta	4PI	8.45 ^a^	6.52 ^b^	6.41 ^b^	7.05 ^ab^	1.010	0.086	0.047	0.002
	8PI	7.22 ^a^	5.66 ^b^	5.29 ^b^	4.80 ^b^	0.657	<0.001	<0.001	0.032
**Lactobacilli**									
Faeces	0	8.66	8.35	8.81	8.78	0.695	0.517	0.255	0.586
	0PI	8.78	9.00	8.97	8.70	1.217	0.956	0.898	0.576
	4PI	9.65	9.87	8.60	9.86	1.381	0.147	0.291	0.306
	8PI	6.82 ^b^	9.05 ^a^	10.06 ^a^	10.04 ^a^	1.031	0.006	<0.001	0.005
Ileal mucosa	4PI	6.12	6.72	7.14	6.30	0.837	0.694	0.334	0.023
	8PI	5.23	5.77	7.33	6.46	1.084	0.666	0.001	0.083
Ileal digesta	4PI	8.52	8.75	8.19	8.58	0.618	0.176	0.279	0.731
	8PI	8.89	9.07	8.98	8.94	0.201	0.346	0.804	0.151
Colonic digesta	4PI	8.84	8.90	8.66	9.10	0.420	0.110	0.929	0.211
	8PI	8.88	8.88	8.84	8.84	0.231	0.964	0.624	0.975
**Ratio lactobacilli:EHEC**									
Faeces	4PI	1.90	−0.038	0.822	1.36	2.4650	0.436	0.860	0.173
	8PI	0.539 ^b^	4.18 ^a^	5.36 ^a^	5.02 ^a^	1.3280	0.002	<0.001	<0.001
Ileal mucosa	4PI	−1.84 ^b^	1.11 ^a^	0.341 ^a^	0.833 ^a^	1.5960	0.006	0.108	0.042
	8PI	−0.199 ^b^	1.63 ^a^	3.11 ^a^	1.96 ^a^	1.0670	0.381	<0.001	0.001
Ileal digesta	4PI	0.728	3.30	2.72	2.34	1.7870	0.100	0.433	0.030
	8PI	3.47	4.79	4.57	4.97	0.6529	0.001	0.012	0.059
Colonic digesta	4PI	0.395 ^b^	2.38 ^a^	2.25 ^a^	2.06 ^a^	1.1560	0.040	0.075	0.014
	8PI	1.66 ^b^	3.23 ^a^	3.55 ^a^	4.04 ^a^	0.6835	<0.001	<0.001	0.038

PRO+/−: presence/absence of probiotic ingredient in diet; PRE+/−: presence/absence of prebiotic ingredient in diet. N = 7 for PRE− and PRO− groups, N = 8 for rest of groups. ^a,b^ indicate statistically significant differences between groups. PI, post inoculation; RSE, residual standard error.

**Table 5:** Numbers of F4 genes in ileal mucosa and colon digesta according to qPCR (in quantifiable animals). Values were measured at day 4 and 8 post inoculation.

Main Effects		PRO−		PRO+			*p*-Value		
		PRE−	PRE+	PRE−	PRE+	RSE	PRE	PRO	Interaction
**Diets**		**CTR**	**scFOS**	**YEA**	**SYN**				
**Log F4 Gene Copies/g FM**	**Day**								
**Ileal mucosa**	4PI	4.36	3.88	4.87	4.43	1.182	0.423	0.456	0.961
	8PI	2.65	3.26	3.50	3.17	0.322	0.518	0.191	0.051
**Colon**	4PI	4.93	4.22	4.42	3.94	1.476	0.231	0.396	0.720
	8PI	3.43	3.79	3.37	3.29	0.443	0.510	0.191	0.299

PRO+/−: presence/absence of probiotic ingredient in diet; PRE+/−: presence/absence of prebiotic ingredient in diet. N = 7 for PRE− and PRO− groups, N = 8 for rest of groups. PI, post inoculation; RSE, residual standard error.

**Table 6:** Effect of experimental treatments on colonic and fecal fermentation. Table includes total short-chain fatty acids (SCFA) (mmol/kg of FM), as well as molar ratio of their components. Values are given for days 4 and 8 post inoculation.

Main Effects		PRO−		PRO+			*p*-Value		
		PRE−	PRE+	PRE−	PRE+	RSE	PRE	PRO	Interaction
**Diets**		**CTR**	**scFOS**	**YEA**	**SYN**				
**Colon**	**Day**								
SCFA (mmol/kg FM)	4PI	80.9	65.4	76.7	84.5	26.3	0.691	0.445	0.238
	8PI	105.9	112.5	118.8	100.7	19.9	0.428	0.933	0.096
**Molar ratio (%)**									
Acetate	4PI	59.6	60.7	61.6	58.3	6.085	0.617	0.918	0.337
	8PI	58.1	57.4	55.5	58.5	3.969	0.441	0.606	0.205
Propionate	4PI	27.0	23.6	24.3	27.7	5.795	0.986	0.746	0.119
	8PI	25.4	25.4	28.4	26.6	3.898	0.531	0.149	0.538
Butyrate	4PI	9.33	10.61	10.16	9.12	2.849	0.910	0.755	0.276
	8PI	12.3	13.1	12.3	9.56	2.925	0.357	0.102	0.107
Valerate	4PI	1.89	2.16	1.75	1.97	0.626	0.287	0.479	0.905
	8PI	2.18	2.09	2.19	2.36	0.641	0.875	0.570	0.587
BCFA	4PI	2.16	2.93	2.24	2.91	1.211	0.116	0.949	0.910
	8PI	1.98 ^xy^	1.69 ^xy^	1.22 ^y^	2.66 ^x^	1.019	0.125	0.773	0.026
**Faeces**									
SCFA (mmol/kg FM)	0PI	82.2	89.4	79.1	73.8	23.04	0.919	0.298	0.487
	8PI	77.4	93.2	88.0	68.2	20.6	0.791	0.340	0.023
**Molar ratio (%)**									
Acetate	0PI	57.2	54.3	60.0	54.5	5.60	0.064	0.501	0.547
	8PI	56.7	56.7	58.4	58.6	3.60	0.601	0.438	0.523
Propionate	0PI	22.8	21.3	19.8	23.8	3.53	0.366	0.858	0.057
	8PI	22.2	21.7	22.3	22.6	2.23	0.973	0.523	0.624
Butyrate	0PI	12.4	15.5	11.3	12.3	3.36	0.119	0.102	0.407
	8PI	11.7	13.6	11.4	10.9	2.32	0.398	0.085	0.174
Valerate	0PI	2.93	3.28	3.20	2.44	0.79	0.514	0.388	0.085
	8PI	2.70	2.89	2.96	2.64	1.02	0.834	0.984	0.522
BCFA	0PI	4.76	5.55	5.77	6.53	1.36	0.146	0.065	0.972
	8PI	4.91	4.82	4.69	4.93	1.18	0.862	0.888	0.698

FM: fresh matter. BCFA: branched-chain fatty acids. PRE+/−: presence/absence of prebiotic ingredient in diet; PRO+/−: presence/absence of probiotic ingredient in diet. N = 7 for PRE− and PRO− groups, N = 8 for the rest of groups. ^x,y^ indicate a statistical trend between groups. PI, post inoculation; RSE, residual standard error.

**Table 7:** Serological levels of TNF-α and Pig-MAP for different experimental treatments expressed as concentrations (pg/mL and g/L, respectively). Values are given for day 0, 4, and 8 post inoculation.

Main Effects		PRO−		PRO+			*p*-Value		
		PRE−	PRE+	PRE−	PRE+	RSE	PRE	PRO	Interaction
**Diets**		**CTR**	**scFOS**	**YEA**	**SYN**				
	**Day**								
**TNFα (pg/mL)**	0PI	124.1	94.5	102.5	109.8	26.40	0.251	0.742	0.063
	4PI	78.7	101.8	100.1	151.9	64.50	0.118	0.135	0.543
	8PI	80.4	85.6	83.0	79.0	18.58	0.932	0.770	0.497
**PigMap (g/L)**	0PI	1.334	0.948	1.112	1.035	0.570	0.269	0.743	0.457
	4PI	1.291	1.809	1.620	1.248	1.385	0.886	0.817	0.380
	8PI	1.653	0.774	0.876	0.823	0.929	0.174	0.286	0.228

PRO+/−: presence/absence of probiotic ingredient in diet; PRE+/−: presence/absence of prebiotic ingredient in diet. N = 7 for PRE− and PRO− groups, N = 8 for rest of groups. PI, post inoculation; RSE, residual standard error.

**Table 8:** Treatment effects on ileal histomorphology measured at 4 and 8 days post inoculation.

Main Effects		PRO−		PRO+			*p*-Value		
		PRE−	PRE+	PRE−	PRE+	RSE	PRE	PRO	Interaction
**Diets**		**CTR**	**scFOS**	**YEA**	**SYN**				
	**Day**								
VH (μm)	4PI	265	257	249	243	45.3	0.656	0.358	0.956
	8PI	298	253	257	284	50.1	0.617	0.760	0.057
CD (μm)	4PI	192	202	179	181	30.7	0.579	0.140	0.699
	8PI	191	198	185	193	25.4	0.402	0.536	0.992
VH:CD	4PI	1.71	1.29	1.43	1.36	0.325	0.442	0.695	0.824
	8PI	1.59	1.29	1.42	1.50	0.332	0.355	0.851	0.124
IEL (cell n°)	4PI	11.6	11.0	10.4	8.41	3.170	0.263	0.107	0.571
	8PI	14.7	13.4	16.1	18.2	4.195	0.793	0.046	0.265
GC (cell n°)	4PI	9.30	8.15	11.4	12.9	5.736	0.952	0.109	0.541
	8PI	13.2	8.02	12.6	9.48	4.561	0.096	0.605	0.169
M (cell n°)	4PI	1.04	1.26	0.766	1.19	0.446	0.056	0.291	0.517
	8PI	0.517	0.835	1.08	1.21	0.460	0.180	0.008	0.585
IEL (cell n°/100 µm)	4PI	4.35	4.30	4.09	3.51	1.001	0.386	0.160	0.464
	8PI	4.85	5.40	6.19	6.54	1.225	0.312	0.009	0.822
GC (cell n°/100 µm)	4PI	3.55	3.19	4.30	5.14	1.912	0.727	0.060	0.389
	8PI	4.45	3.11	3.90	3.46	1.605	0.136	0.866	0.443
M (cell n°/100 µm)	4PI	0.551	0.648	0.426	0.664	0.254	0.079	0.543	0.450
	8PI	0.290	0.424	0.595	0.634	0.258	0.360	0.010	0.612

Measured parameters: villous height (VH); crypt depth (CD); ratio villous height:crypt depth (VH:CD); intraepithelial lymphocytes (IEL); goblet cells (GC); mitosis (M). PRO+/−: presence/absence of probiotic ingredient in diet; PRE+/−: presence/absence of prebiotic ingredient in diet. N = 7 for PRE− and PRO− groups, N = 8 for rest of groups. PI, post inoculation; RSE, residual standard error.

## Data Availability

The data presented in this study are available on request from the corresponding author. The data are not publicly available due to their proprietary nature.
